# Noninvasive Combined Diagnosis and Monitoring of *Aspergillus* and *Pseudomonas* Infections: Proof of Concept

**DOI:** 10.3390/jof7090730

**Published:** 2021-09-06

**Authors:** Radim Dobiáš, Anton Škríba, Tomáš Pluháček, Miloš Petřík, Andrea Palyzová, Marcela Káňová, Eva Čubová, Jiří Houšť, Jiří Novák, David A. Stevens, Goran Mitulovič, Eva Krejčí, Petr Hubáček, Vladimír Havlíček

**Affiliations:** 1 Department of Bacteriology and Mycology, Public Health Institute in Ostrava, 702 00 Ostrava, Czech Republic; radim.dobias@zuova.cz (R.D.); eva.krejci@zuova.cz (E.K.); 2 Department of Biomedical Sciences, Faculty of Medicine, University of Ostrava, 703 00 Ostrava, Czech Republic; 3 Institute of Microbiology of the Czech Academy of Sciences, 142 20 Prague, Czech Republic; anton.skriba@biomed.cas.cz (A.Š.); tomas.pluhacek@biomed.cas.cz (T.P.); palyzova@biomed.cas.cz (A.P.); jiri.houst@biomed.cas.cz (J.H.); jiri.novak@biomed.cas.cz (J.N.); 4 Department of Analytical Chemistry, Faculty of Science, Palacký University, 771 46 Olomouc, Czech Republic; 5 Institute of Molecular and Translational Medicine, Faculty of Medicine and Dentistry, Palacky University, 779 00 Olomouc, Czech Republic; milos.petrik@upol.cz; 6 Department of Anesthesiology and Intensive Care Medicine, University Hospital Ostrava, 708 00 Ostrava, Czech Republic; marcela.kanova@fno.cz; 7 Institute of Physiology and Pathophysiology, Faculty of Medicine, University of Ostrava, 701 03 Ostrava, Czech Republic; 8 Department of Intensive Medicine, Emergency Medicine and Forensic Studies, University of Ostrava, 701 03 Ostrava, Czech Republic; 9 Department of Internal Medicine, Ostrava City Hospital, 728 80 Ostrava, Czech Republic; cubova.eva@gmail.com; 10 Infectious Disease Research Laboratory, California Institute for Medical Research, San Jose, CA 95128, USA; stevens@stanford.edu; 11 Division of Infectious Diseases and Geographic Medicine, Stanford University School of Medicine, Stanford, CA 95128, USA; 12 Clinical Department of Laboratory Medicine Proteomics Core Facility, Medical University of Vienna, A-1090 Wien, Austria; goran.mitulovic@meduniwien.ac.at; 13 Department of Medical Microbiology, 2nd Faculty of Medicine, Charles University and Motol University Hospital, 150 06 Prague, Czech Republic; petr.hubacek@fnmotol.cz

**Keywords:** *Aspergillus fumigatus*, *Pseudomonas aeruginosa*, invasive infection, noninvasive diagnosis, coinfection, virulence factor, siderophores, quorum-sensing molecules

## Abstract

In acutely ill patients, particularly in intensive care units or in mixed infections, time to a microbe-specific diagnosis is critical to a successful outcome of therapy. We report the application of evolving technologies involving mass spectrometry to diagnose and monitor a patient’s course. As proof of this concept, we studied five patients and used two rat models of mono-infection and coinfection. We report the noninvasive combined monitoring of *Aspergillus fumigatus* and *Pseudomonas aeruginosa* infection. The invasive coinfection was detected by monitoring the fungal triacetylfusarinine C and ferricrocin siderophore levels and the bacterial metabolites pyoverdin E, pyochelin, and 2-heptyl-4-quinolone, studied in the urine, endotracheal aspirate, or breath condensate. The coinfection was monitored by mass spectrometry followed by isotopic data filtering. In the rat infection model, detection indicated 100-fold more siderophores in urine compared to sera, indicating the diagnostic potential of urine sampling. The tools utilized in our studies can now be examined in large clinical series, where we could expect the accuracy and speed of diagnosis to be competitive with conventional methods and provide advantages in unraveling the complexities of mixed infections.

## 1. Introduction

*Aspergillus fumigatus* is an opportunistic fungal pathogen, the most lethal fungal pathogen [1,2], and, as recently noted, the most common cause of pulmonary mycosis associated with coronavirus disease (COVID-19) [3]. Delineating aspergillosis infection from colonization is difficult, and better tools are needed [4]. Among bacterial pathogens, *Pseudomonas aeruginosa* is a leading cause of acute nosocomial infections and pneumonia in particular (45%), with a high mortality rate, ranging from 13% to 50% [5]. *P. aeruginosa* is replete with a diverse arsenal capable of activating, modifying, and destroying host defense mechanisms [6]. These weapons can be present concurrently with multidrug resistance, magnifying the risks posed by *P. aeruginosa* [7]. Fungi, particularly *Aspergillus*, and *Pseudomonas* can co-exist, interact, and likely compete in critically ill patients with invasive disease. Recently, the problem has been magnified by the susceptibility of critically ill COVID-19 patients in intensive care units (ICUs) to opportunistic fungal and bacterial infections. Early diagnosis is crucial. In the present study, we show mass spectrometry tools can, noninvasively and with sensitivity, diagnose these infections, even when these microbes are concurrently present, and monitor their course. This is demonstrated with five intensive care patients and the use of rat infection models.

The acquisition of iron is mandatory for the growth of these pathogens [8]. Our strategy is centered on detecting the metallophores secreted by these pathogens, and finding them in the invasive stage of disease, particularly as iron conjugates. Thus, they can serve as infection biomarkers. Characteristic isotopic profiles of the metals in mass spectra can be used for specific detection of metallophores in patients’ bodily fluids. Our approach involves the combined use of liquid chromatography (LC)–electrospray (ESI) and matrix-assisted laser desorption ionization (MALDI) mass spectrometry (MS) with isotopic data filtering. State-of-the-art data were collected using Fourier transform ion cyclotron resonance (FTICR), currently the only analytical technique capable of unequivocally detecting and characterizing pathogen-related metal-containing compounds in host body fluids. The physicochemical background of isotope filtration of mass spectrometry data has been described in a recent publication [9]. In addition to metallophores [10,11], pathogens’ autoinducers during quorum sensing (QS) can also be used as specific early markers of invasive infections.

In human serum, the iron transport protein transferrin maintains free ferric iron concentrations at far too low (approximately 10^−24^ M) to support *P. aeruginosa* growth and proliferation [12]. However, *P. aeruginosa* has mechanisms that enable it to compete for iron with hosts and filamentous fungi in the microbiome under low and high iron conditions [13]. These include secretion of siderophores—pyoverdines (Pvds) and pyochelin (Pch)—which are major virulence factors of the pathogen [14]. In the competition for iron, *A. fumigatus* likewise needs to capture and internally utilize iron, and does so by utilizing its own siderophore machinery [8,15]. In addition, *P. aeruginosa* can switch from iron-denial-based (Pvd) to toxin-based (pyocyanin) antifungal activity when combating *A. fumigatus* [16].

The complex *P. aeruginosa* metallophore machinery is accompanied by a highly tuned QS system [17]. One of the earliest QS components is the alkylquinolone, 2-heptyl-4-quinolone (HHQ). HHQ activates the PqsR multiple transcriptional virulence factor regulator, thereby driving the production of QS molecules, toxins, and biofilm formation that stimulate host cells (represented by macrophages in Supplementary Materials Figure S1) and promote tissue damage during *P. aeruginosa* infections [18].

In vitro studies have shown that secreted siderophores and small QS molecules play essential roles in pathogenesis, so they are appropriate targets for next-generation diagnostic, therapeutic tools in various applications, including critical care medicine [6]. Here, we illustrate the potency of these applications by demonstrating the noninvasive detection of siderophores (Pvds, Pch) and the quorum-sensing molecule HHQ as early markers of invasive *P. aeruginosa* infections in breath condensate and urine samples from five critically ill patients. Two cases were fatal. The first case was associated with *A. fumigatus* coinfection, demonstrated by the presence of its triacetylfusarinine C (TafC) siderophore. In the second fatal case, severe lung fibrosis in COVID-19 pneumonia was combined with *P. aeruginosa*, *Candida glabrata*, and Herpes simplex virus infections. All pathogens were authenticated in clinical specimens by standard culture, microscopy, matrix-assisted laser desorption with time of flight (MALDI-TOF) mass spectrometry, serum antigen, and nucleic acid detection.

## 2. Materials and Methods

### 2.1. Culture, Serology and MALDI-TOF

Fungal and bacterial strains were identified by MALDI-TOF mass spectrometry with a Biotyper instrument (Bruker Daltonik, Bremen, Germany). Early fungal biomarkers were measured during the patients’ hospitalizations. Galactomannan (GM) antigen was detected with *Aspergillus* EIA (Bio-Rad Platelia™, Marnes-la-Coquette, France) following the manufacturer’s instructions. The serum samples were considered positive if the GM index value was ≥1 following the Probable Invasive Pulmonary Mold Diseases criteria by the European Organization for Research and Treatment of Cancer and the Mycoses Study Group Education and Research Consortium [19]. 1,3-β-d-glucan (BDG) was determined with the Fungitell assay (Associates of Cape Cod, Falmouth, MA, USA); patient serum results were considered positive if the BDG concentration was ≥80 pg/mL. *A. fumigatus*-specific antibodies (IgA, IgG) were detected using *A. fumigatus* IgG/IgA ELISA kits (Immunolab GmbH, Kassel, Germany) according to the manufacturer’s instructions. The serum samples were considered positive if the index values of both IgG and IgA classes were ≥1.2. The test allowed separate determination of specific antibody reactivities for IgA and IgG isotypes.

### 2.2. Liquid Chromatography and Mass Spectrometry Assay for Biomarker Monitoring

Desferrioxamine E (FoxE), ferricrocin (Fc), TafC, Pch, and a ferriform of PvdE/PvdD were obtained from EMC Microcollections GmbH (Tübingen, Germany), and HHQ from Sigma-Aldrich (Prague, Czech Republic). Breath condensates were obtained as described [20]. Urine, serum, breath condensate, endotracheal aspirates, and lung tissue samples were subjected to two-step liquid–liquid extraction [21]. Briefly, 50 µL samples were spiked with a FoxE internal standard (1 µg/mL, 7.5 µL), extracted twice with ethyl acetate, and dried under reduced pressure. The remaining aqueous phase was mixed with four volumes of methanol and frozen (−80 °C, 1 h). Precipitated proteins were removed by centrifugation (14,000× *g*, 4 °C, 10 min), and the supernatant transferred to a vial with the residue from the evaporated ethyl acetate fraction and concentrated under reduced pressure. The pooled extract was resuspended in 15% liquid chromatography–mass spectrometry-grade acetonitrile (ACN), and then subjected to high-performance liquid chromatography (HPLC)–mass spectrometry.

The urine samples from the mixed infection experiments in rats (Rats 3–7) were analyzed by an Acquity M-class HPLC system connected to a Synapt G2-Si Q-TOF mass spectrometer (Waters Corporation, Manchester, UK). Each sample (1 µL) was injected in triplicate (rat urines) or single analysis (human patient aspirates) onto an Acquity HSS T3 C18 analytical column (1.8 μm, 1.0 × 150 mm, Waters Corporation, Manchester, UK). Gradient elution was performed at a 50 µL/min flow rate: 0 min (3% B); 2 min (3% B); 8 min (50% B); 8.1 min (99% B); 12 min (99% B); 12.1 min (3% B); 15 min (3% B). Solvent A contained 0.1% formic acid in the water, and solvent B contained 0.1% formic acid in acetonitrile. The spectrometer was operated in positive-ion electrospray mode within the 200–1500 Da mass range.

For sensitivity enhancement, the remaining samples (Rats 1, 2, and all patients) were analyzed by a Dionex UltiMate 3000 HPLC system (Thermo Fisher Scientific, Waltham, MA, USA), equipped with the same HPLC column but coupled to a SolariX 12T FTICR (Bruker Daltonik, Bremen, Germany). Portions of 2 μL of the solution were injected into the system. Then, the analytes were eluted using a mobile phase with a 50 μL/min flow rate, starting with isocratic 2% B for 2 min, with linear increases from 2 to 60% and 60–99% from 2 to 9 min and 9 to 11 min, respectively, followed by a 99% B isocratic wash from 11 to 14 min, a return to 2 % B between 14 and 14.5 min, and then re-equilibration with 2% B for 5.5 min. Here, B is 95% ACN with 0.1% formic acid in the water, and the A solvent is 1% ACN with 0.1% formic acid in water. The spectrometer was operated in positive-ion mode with quadrupole settings of 200–600 and 500–1000 Da, in both cases with the appropriate ion transfer optics tuning. Rat samples were analyzed in triplicates and patient samples were analyzed once or in duplicates. Data were processed qualitatively and quantitatively by our inhouse CycloBranch [9] and Data Analysis 5.0 software (Bruker Daltonik, Bremen, Germany), respectively.

### 2.3. Biomarker Standardization and Quantitation in Liquids and Tissues

Urine calibration standards were prepared from 50 µL portions of pooled control uninfected human urine (10 donors) spiked with commercial PvdE, Pch, HHQ, TafC (variable concentration, 15 µL), and FoxE (1 µg/mL, 7.5 µL) as an internal standard. An EMC Microcollections PvdE PAO1 standard isolated from *P. aeruginosa* ATCC 15692 contained a mixture of PvdE and PvdD. Quantification statistics, including the limit of detection (LOD) and limit of quantitation (LOQ), were obtained for the sum of these two Pvds, then calculated for all three Pvds (D, E, C) recorded in human and rat samples. The rat serum Pvd calibration curve (serum standard obtained from Sigma-Aldrich, Prague, Czech Republic) was constructed with the same protocol. The LOD and LOQ values were calculated from the standard deviation of the intercept multiplied by 3.3 and 10, respectively. All samples were analyzed in triplicate, and results are expressed as the means ± standard deviation (SD). The instrumental performance was checked by a system suitability test using the HPLC peptide standard mixture (Sigma-Aldrich, Prague, Czech Republic). The LOD values were 1.10, 2.10, 0.06, and 0.27 ng/mL for urine concentrations of Pvds, Pch, HHQ, and TafC, respectively. Serum total Pvd (D, E) LOD was 0.4 ng/mL. All calibration curves can be found in Supplementary Materials Figures S2 and S3.

The rat extracts of lung homogenates were prepared by standard addition of Pvd to each extract. The lung tissue was cut in a cryostat, placed in a 1 mL vial, and dry-weighed. Then, a H_2_O:methanol (1:1) mixture (200 µL) and FoxE (1000 ng/mL, 7.5 µL) were added, shaken for 1 h at 1000 rpm, at 6 °C, and centrifuged at 14,000× *g* at 4 °C for 10 min. The supernatant was stored, but the tissue precipitate was subjected to an additional ethyl acetate extraction (2 × 200 µL) by mixing with vortex agitation for a minute. The extract was combined with the aqueous methanol extract, evaporated to dryness, and redissolved in 400 µL of 5% ACN before HPLC analysis. PvdE was quantified using the standard addition method with two additions (50 and 100 ng/mL Pvd). The mixed-infection model (see Section 2.4) calibrations, LODs, and LOQs are depicted in Supplementary Materials Figure S4.

To assist the assessment of the infected rat samples, for standardization, background Pvd and Pch signals in rats from *P. aeruginosa* inoculum (100 µL) were determined in two biological replicates. The extraction and processing of 10^8^ CFU/mL, the inoculation equivalent loaded into rat lungs, provided a Pvd signal between the instrument LOD and LOQ, representing less than 0.1% of the final rat infection values. A high maximum Pch background contribution (75 ng/mL) was calculated from normal rat urine (3.3 mL/100 g body weight per day). Pvd, Pch, and HHQ have not been previously reported to be synthesized by mammalian hosts. For Pvd and HHQ stability and HHQ fragmentation behavior, see the Supplementary Materials Figures S5 and S6, respectively.

### 2.4. Animal Experiments

Animal studies of siderophore distribution were performed using 10-week-old female Lewis rats (Envigo, Horst, The Netherlands). Two immunocompetent rats were infected intratracheally with *P. aeruginosa* ATCC 15692 (10^8^ CFU in 100 µL) under 2% isoflurane anesthesia (Forane, Abbott Laboratories, Abbott Park, IL, USA), as previously described (Rats 1 and 2, Table 1). Five hours post-infection, the animals were sacrificed, and serum and urine samples were collected and frozen (−80 °C). The lungs from one rat were excised, dried, and weighed. The sample was homogenized by milling with glass beads (1 mm diameter) in methanol (1 h). The beads were decanted with methanol twice, and the extracts were pooled and dried. Two other noninfected animals provided a control group.

In addition to the mono-infection *P. aeruginosa* model, the mixed *A. fumigatus*–*P. aeruginosa* model with immunocompromised rats was used. The previously reported protocol of invasive aspergillosis in rats [22] was amended with the intramuscular application of *P. aeruginosa* (Rats 3–7, Table 2). Briefly, five rats were intratracheally infected with *A. fumigatus* ATCC 46645 (10^8^ CFU in 100 μL). Forty-eight hours later, *P. aeruginosa* ATCC 15692 (10^9^ CFU in 100 µL) was injected into their thigh muscles of the left hind legs. Three other noninfected animals were used as a control group. The experiment was terminated 5 h post-infection with *P. aeruginosa*. The urine from experimental animals of mixed infection was collected twice a day starting 1 day before the *A. fumigatus* inoculation until the end of the experiment.

## 3. Results

### 3.1. A. fumigatus and P. aeruginosa in Co-Infection

**Figure 1:**
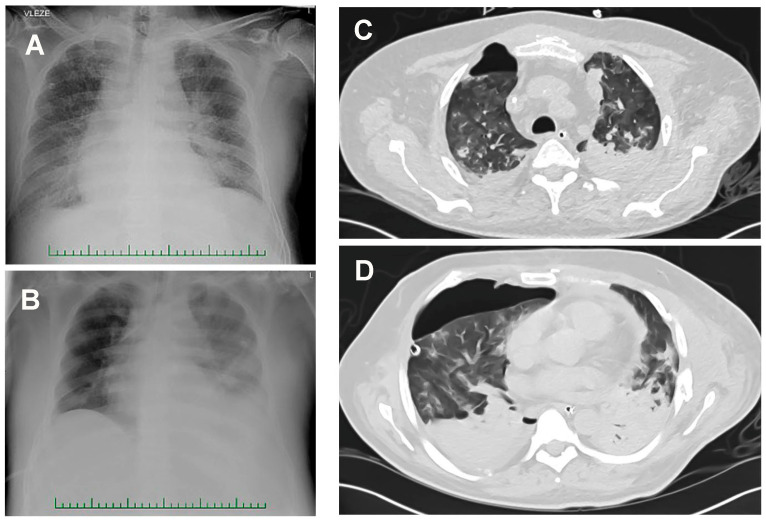
ARF patients’ chest radiography and CT scans. (**A**,**B**) Radiographs showing increasing bilateral, inhomogeneous blurring, likely caused by inflammatory infiltrations (Patient 1). (**C**,**D**) Atelectasis in both lower lung lobes and irregular multiple ground glass opacities revealed by the CT scan of Patient 2. Right-sided pneumothorax and small amount of fluid in bilateral pleural cavities are also discernable.

Patient 1 (man, age 65), with cirrhosis and multiorgan dysfunction (serum creatinine 950 μmol/L), was transferred from a medical inpatient unit to an ICU owing to sepsis (C-reactive protein 211 mg/L, procalcitonin 99.8 ng/mL). He required mechanical lung ventilation, vasopressors, and continual venovenous hemodialysis. He was treated with cefotaxime because of repeated aerobic hemocultures positive for *E. coli*. Hemolytic *E. coli* was also present in the urine, but susceptible to antibacterial therapy (Eucast v10, https://eucast.org, accessed on 15 December 2020). Due to diarrhea episodes, the patient was given metronidazole empirically. When acute respiratory failure (ARF) developed, the patient was intubated, connected to a ventilatory support system, and continuously dialyzed. Chest radiography showed increasing bilateral, inhomogeneous blurring, believed to be caused by inflammatory infiltrations (Figure 1A,B).

**Figure 2:**
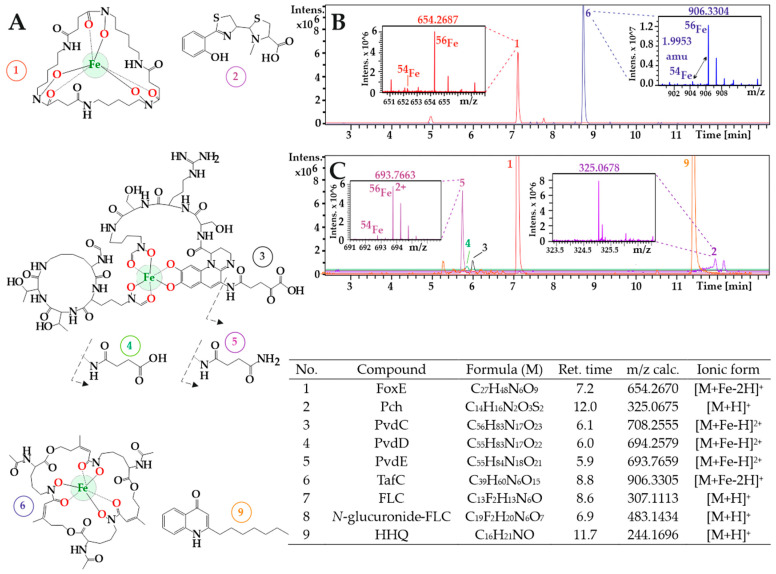
Noninvasive detection of *Aspergillus* and *Pseudomonas* biomarkers returned by CycloBranch. (**A**) Chemical structures of the siderophores (1–6) and HHQ (9). Compound numbering is described in the associated table. (**B**) Patient 1’s urine profile compiled from reconstructed ion chromatograms recorded in the low- and high-mass experiments (see Materials and Methods for details). TafC was detected as a singly charged species (m/z 906.3304, see the right inset for Peak 6, indicating the presence of ^54^Fe and ^56^Fe stable isotopes). The left inset (Peak 1) indicates the isotopic profile of FoxE. (**C**) Patient 1’s breath condensate profile sampled on the same Day 12 of illness with a mixture of doubly charged pyoverdines. Peak 5 in the left inset is represented by an isotopic pattern, indicating two charges (^54^Fe/^56^Fe isotopes are separated by approximately one Dalton). The right inset belongs to pyochelin singly protonated molecule. M in the table stands for neutral molecule; Ret. time, retention time. Fluconazole and its glucuronide structures are reported in Supplementary Materials Figure S8.

On Day 6 of hospitalization, after intubation, carbapenem-resistant (CaR) but cephalosporine-, amikacin-, and colistin-susceptible *P. aeruginosa* was detected for the first time in sputum, along with new accompanying flora of yeasts and hyphae with the typical 45° branching angle of *A. fumigatus*, which was subsequently defined by culture microscopy and a serum GM test. *Enterococcus faecium* was noted on Day 10 in aerobic hemoculture. Both *P. aeruginosa* and *E. faecium* were also detected on the next day in sputum. Day 11 was the first breath condensate mass spectrometry sampling day and it was negative for *P. aeruginosa* and *A. fumigatus*. Serum examination (Day 11) revealed high BDG levels (488 pg/mL). On the same day, GM and *A. fumigatus*-specific IgA were detected in the serum (with GM and IgA index positivities of 1.076 and 1.7, respectively), and the presence of *A. fumigatus* was confirmed by culturing from sputum four days later. After 72 h of continuous hemodialysis, the patient was still oliguric, so a further two sessions were provided. Due to the patient’s oliguria, only a single urine sample of unclear quality was obtained on the same Day 12 (Figure 2B). In contrast, three breath condensate samplings were obtained on Days 11, 12, and 17. PvdE, Pch, and HHQ (Figure 2A) were detected by MS (at 85.3 ± 3.4, 135.1 ± 4.2, and 12.9 ± 1.2 ng/mL, respectively) in the breath condensate obtained on Day 12 (Figure 2C). No Pvds, Pch, or HHQ were detected in the patient’s urine sampled on Day 12 (Table 1). However, evidence of invasive *A. fumigatus* coinfection (Table 2) was detected in the urine by the presence of its siderophore, TafC (23 ± 1 ng/mL).

Voriconazole treatment (Day 14) was accompanied by decreased serum BDG levels (to 441 pg/mL) and there was a marked decrease in GM (to 0.444), indicating cessation of *A. fumigatus* growth or reduction of infection burden. On the 15th day, the GM level had further decreased, and the *A. fumigatus*-specific IgA index had increased to 2.4. Aerobic cultivation of bacteria in sputum obtained on Day 17 revealed resistant *Serratia marcescens* and *Elizabethkingia miricola*. Finally, there was a gradual deterioration of the circulatory parameters with an increase in the consumption of vasopressors, asystole, and circulatory failure, resulting in death on the 19th day after the start of hospitalization. Clinical observations made during the hospitalization are depicted in the Supplementary Materials Figure S7.

### 3.2. New Urine Biomarkers of Pseudomonas aeruginosa

Patient 2 (man, age 42), first admitted to ICU following a suicide attempt, suffered from polytrauma (spine, pelvis, chest, and femur) and hemorrhagic shock. After resuscitation, he was treated with massive transfusion, a high dose of vasopressors (norepinephrine), mechanical lung ventilation, and drainage for pneumothorax. Despite intensive empirical antibiotic treatment (tigecycline, levofloxacin), severe pneumonia developed into ARF before a re-admission to the ICU and institution of extracorporeal membrane oxygenation (Days 32–38). A CT scan of the patient’s chest showed increasing bilateral, inhomogeneous blurring, likely caused by inflammatory infiltrations, atelectasis in the lower lung lobes, and irregular multiple ground-glass opacities in other parts of the lung (Figure 1C,D).

The ICU staff dealt with new attacks of sepsis [23], represented with increased fluid requirements, need for vasopressor support, positive end-expiratory pressure on artificial ventilation, and the rise of proinflammatory cytokines. In addition to several cultures of *Stenotrophomonas maltophilia* and *Enterococcus faecalis*, CaR *P. aeruginosa* was first cultivated from sputum on Day 44, despite applications of antibiotics (levofloxacin, tigecycline, sulfamethoxazole, and meropenem). Fluconazole (FLC) had been used in prophylaxis, and micafungin in pre-emptive antimycotic therapy due to increasing serum BDG levels (to 285 pg/mL), the possibility of ICU-acquired opportunistic fungal lung invasion, and the risk of *Candida* infection. *Pseudomonas aeruginosa* was repeatedly cultivated from sputum on Day 51, and the patient was treated with colistin.

On Day 57, a mixture of Pvds, dominated by PvdC at 82.7 ± 0.3 ng/mL, was secreted into the host urine in their ferriforms (Supplementary Materials Figure S8). Pch and HHQ were identified by CycloBranch [9] as protonated molecules at 90.8 ± 1.1 and 1.4 ± 0.5 ng/mL, respectively (Table 1). Cultures on Day 60 indicated both vancomycin-resistant *E. faecium* and CaR *P. aeruginosa* in urine and *E. faecalis* alone in sputum. Colistin therapy was continued, the severe bacterial infection was treated successfully, and the patient was released from the ICU.

### 3.3. Animal Infection Models

The experiments in a rat *P. aeruginosa* lung mono-infection model showed high Pvds and Pch levels (>10 μg/mL for PvdE and >1 μg/mL for Pch) in the urine five hours post-inoculation. In contrast, the levels of monitored serum Pvds did not exceed 0.3 μg/mL. The Pch values were very low. The initial Pvd contribution from the inoculate was negligible, in contrast to that of Pch (Table 1).

In the *A. fumigatus/P. aeruginosa* coinfection model, both pathogens were clearly marked by their respective siderophores (Table 2) in three animals (Rats 3, 6, and 7). In the remaining two infected animals, either *P. aeruginosa* (Rat 4) or *A. fumigatus* (Rat 5) mono-infection biomarkers were detected. Rat 5 was sacrificed early (2 h) after *P. aeruginosa* infection due to poor health, which could explain the absence of siderophores produced by *P. aeruginosa* in the urine sample.

### 3.4. Pseudomonas aeruginosa in COVID-19-Associated Pneumonia

The SARS-Cov-2 pandemic has greatly increased the frequency of opportunistic fungal, bacterial, and mixed fungal–bacterial infections, particularly in ICU units. Rapid diagnosis is critical. In a subset of COVID-19 patients with pneumonia, the diagnostic tools described in this report were applied. We report three individuals (Patients 3–5) in which mixed bacterial–fungal infections were documented or suspected. HHQ and Pch were detected in their endotracheal aspirates. All analyses were carried out in two technical replicates (Table 1). No bacterial pyoverdines were detected.

Patient 3 (man, age 66) was first admitted to ICU due to respiratory insufficiency with COVID-19 pneumonia and further progression despite the aggressive artificial ventilation regime. He required extracorporeal membrane oxygenation (ECMO) therapy on Day 3. Simultaneously, piperacillin–tazobactam therapies with acyclovir were initiated. On Day 10, *P. aeruginosa* in airway aspirate was rarely detected; therefore, amikacin was added. Due to a suspected ventricle thrombus, anticoagulation treatment with heparin and acetylsalicylic acid was initiated in addition to piperacillin–tazobactam therapy. Shortly after ECMO start, the patient’s clinical status improved and stabilized. Unfortunately, on Day 12 the patient developed uncontrolled bleeding combined with disseminated intravascular coagulopathy. The ECMO was terminated, and intravascular catheters were extracted. Shortly after, the coagulation parameters improved, and the bleeding stopped. The next day, massive amounts of *P. aeruginosa* (10^7^ cells per 1 mL of ETA) were detected (Table 1), and inhaled colistin initiated. Due to constant critical respiratory insufficiency, aggressive artificial ventilation was continued, with slow improvement. On Day 35, the patient was oriented. The patient further clinically improved and survived.

Patient 4 (man, age 77) was admitted to the hospital after one week of fevers and two days of respiratory symptoms. Due to rapid clinical deterioration, artificial ventilation and cefazoline prophylaxis was initiated. Owing to bloodstream *Staphylococcus aureus* infection on the next day, linezolid treatment was added. After clinical improvement, the antibiotic therapy was upgraded to linezolid with ciprofloxacin. On Day 13, massive hemoptysis was observed, and aspiration pneumonia developed. Three days later, catheter sepsis with *Klebsiella pneumonia*, *Burkholderia multivorans*, and multiresistant *P. aeruginosa* in ETA and in urine (10^3^/mL) were noted (Table 1). Upon therapy switch to voriconazole and trimethoprim/sulfamethoxazole, the patient improved and survived.

Patient 5 (woman, age 62) was admitted to the hospital after 10 days of fever and progressive shortness of breath due to COVID-19 pneumonia. Shortly after respiratory insufficiency progressed, mechanical ventilation and empirical therapy with ampicillin-sulbactam, acyclovir, and remdesivir started. On Day 3, the antimicrobial therapy was modified according to the susceptibility testing results; thus, following ETA cultures of *K. pneumonia*, *P. aeruginosa* (Table 1), *Candida albicans*, *Pneumocystis jirovecii*, and Herpes simplex virus, FLC and trimethoprim–sulfamethoxazole were added. Shortly after, antibiotic therapy was switched to piperacillin–tazobactam instead of ampicillin–sulbactam. Later, the antibiotics were changed to meropenem, intravenous ciprofloxacin, and inhaled colistin. *C. glabrata* was cultured from ETA, and anidulafungin substituted for FLC. Despite the aggressive therapy, the clinical status worsened. On Day 10 of hospitalization, antibiotic therapy was changed to acyclovir, meropenem, voriconazole, trimethoprim–sulfamethoxazole, and rifampicin. The patient died on Day 15 after ICU admission.

## 4. Discussion

**Figure 3:**
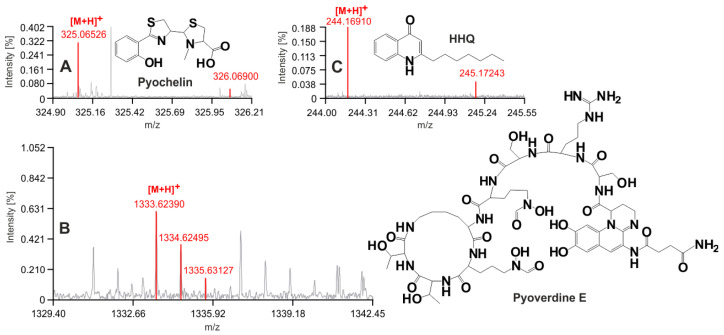
MALDI-FTICR-MS annotation of Patient 1’s breath condensate (sampled Day 12). Pch (**A**), PvdE (**B**), and HHQ (**C**) were all detected by MALDI MS as protonated forms. The structures of these molecules are shown. The annotation in red was returned by CycloBranch software [9] when running the MALDI mass spectrum against the library of *P. aeruginosa* secondary metabolites. The red color was added to an examined spectrum by the software automatically. The color indicates which isotope features have been used by the computing algorithm for the annotation process.

The key timeframe for diagnosing both the infection and the pathogen, and thus planning the antimicrobial intervention, is the window of opportunity when pathogen proliferation accelerates, for which specific, sensitive biomarkers are urgently required (Figure 3). When there are mixed infections, particularly mixed bacterial–fungal infections, as is common, being able to sort through the ensuing diagnostic complexities in a timely fashion in the face of a synergistic danger to patients, is a formidable problem.

Mass spectrometry approaches can be applied in hospital labs with Biotyper-like facilities. For example, aromatic Pvds, Pch, and HHQ can be readily analyzed by MALDI-MS. This is especially true when analyzing samples with relatively low chemical complexity, such as breath condensates. PvdE, Pch, and HHQ were directly detectable in our patient samples by MALDI-FTICR-MS (Figure 3). Furthermore, degradation rates of all the detected siderophores were slow during storage (37 °C) in model human serum and Patient 2’s urine, and degradation was compensated by PvdD gain over three days at body temperature storage (Supplementary Materials Figure S5). The conversion of succinamide to succinic acid in Pvds (the conversion of PvdE to PvdD) was faster in urine than in serum (Supplementary Materials Figure S5A,B). PvdC was stable in both urine and serum, but not Pch, whose metabolic and secretion fates remain unknown. HHQ was very stable in urine, but not in serum, in which 60% of the original ion signal was lost during a week of storage (Supplementary Materials Figure S5).

Experiments with the animal *P. aeruginosa* mono-infection model suggest that detecting siderophores are more practical and sensitive as urine biomarkers than serum biomarkers. Their urine levels in infected rats were two orders of magnitude higher than those in sera (Table 1, mono-infection model). Although the serum total Pvd LOD and LOQ were 0.4 ng/mL and 1.2 ng/mL, respectively (lower than corresponding limits in urine), breath condensate and urine are better analytical bodily fluids due to concentration effects, their less complex chemical nature, and because they are more easily obtained. The tissue content of Pvd in infected lungs of the model rats was in the ng/mg range. Of note, the higher Pvd and Pch concentration levels detected in the mixed infection model could arise from the high (10^9^ CFU) inoculation load we used (Table 2).

The invasive endotracheal sampling was used in three COVID-19 patients with *Pseudomonas* pneumonia. HHQ and Pch, but not pyoverdines, were detected in their ETAs. We may speculate whether the absence of Pvd in the aspirates was caused by the respective antibacterial therapies, by an excessive iron load available in the COVID-19-deteriorated lungs, by an early onset of the bacterial infection, by lower stress within the lung microbiome, or by combination thereof. On the other hand, the production of Pch and HHQ was observed in all patients (1–5). These preliminary data must be confirmed in future clinical trials for which ETA samples handling is fully optimized.

## 5. Conclusions

Our detection of incipient proliferation of *P. aeruginosa* and *A. fumigatus* by monitoring specific biomarkers in patients’ urine and breath condensates demonstrates that mass spectrometry permits timely interventions. State-of-the-art data were collected using Fourier transform ion cyclotron resonance mass spectrometry, the research analytical technique capable of unequivocally detecting and characterizing the pathogen-related metal-containing compounds in host body fluids. Direct monitoring of microbial virulence factors such as HHQ, Pvd, Pch, and TafC may enable better and timelier antibacterial and antimycotic interventions than would otherwise be possible.

Our detection of HHQ, a quorum-sensing molecule in the breath condensate, ETA, or urine of critically ill patients, is the first observation demonstrating incipient *P. aeruginosa* invasion in vivo. Our approach also enables quantification of the microbial siderophores Pvds, Pch, and TafC at the ng/mL concentrations. Moreover, assessing these siderophores’ stability and partitioning between serum, urine, and breath condensate may enable the future definition of biomarker breakpoints for critical human specimens.

The observations in these five patients, and our demonstrations in a rat model of the sensitivities of our techniques, show the potential of practical applications of our technologies amidst the complexities of single and mixed opportunistic infections in the clinical setting. This will now require extensive clinical trials, and we would expect enhanced speed and superior definition of the pathogens when our technologies are compared to current conventional diagnostics (e.g., specimen cultures, GM assays, PCR, BDG, etc.).

There is still a lot of work ahead in diagnosing bacterial and fungal coinfections. In the present studies, some of the samples were provided later than we would have liked, for the purpose of optimal detection, and, as a result, could have given a weaker signal. The molecular distribution kinetics and biomarker fate during circulation in the patients’ bodies have presently been understudied. For this purpose, mixed infection animal models must be further developed. The biomarker breakpoint definitions, and the timing of their appearance, must be defined in clinical trials, which will require the prospective collection of specimens and their testing. The specificity of metallophore or quorum-sensing molecule secretion by pathogens should be assessed. We hope that applying diagnostic tools to the secondary metabolism of pathogens may open a new analytical field in microbial diagnostics.

## Figures and Tables

**Table 1:** Distribution of Pvds, Pch, and HHQ in humans and rats reflects *Pseudomonas* infection. Urine and breath condensate were collected from two patients on the indicated day. In the rat mono-infection model, two animals were inoculated with *P. aeruginosa* cells, and samples of their urine, serum, and tissues were collected five hours post-inoculation. Background Pvd signal in the inoculum ** (mono-infection model, sample Blank) was negligible, in contrast to Pch. * Quantified based on single-point calibration; DET, detected (value between LOD and LOQ); -, not detected; ETA, endotracheal aspirate; ^&^ Standard deviation is calculated from the measurement precision of the corresponding matrix-matched calibrators.

Sample		Concentration (ng/mL)				
	Specimen	PvdE	PvdD	PvdC	Pch	HHQ
Patient 1	condensate	85.3 ± 3.4	DET	12.0 ± 0.8	135.1 ± 4.2	12.9 ± 1.2
Patient 2	urine	23.7 ± 0.1	-	82.7 ± 0.3	90.8 ± 1.1	1.4 ± 0.5
Patient 3	ETA	-	-	-	19.5 ± 0.9 ^&^	1.4 ± 0.1 ^&^
Patient 4	ETA	-	-	-	24.2 ± 1.1 ^&^	0.2 ± 0.01 ^&^
Patient 5	ETA	-	-	-	252.4 ± 2.8 ^&^	1.0 ± 0.04 ^&^
Rat 1	urine	14,334 ± 352	102.6 ± 3.7	390.7 ± 13.1	1233.9 ± 26.3	-
	serum	53.0 ± 3.0	3.4 ± 0.2	-	-	-
Rat 2	urine	12,366 ± 843	74.3 ± 4.1	261.4 ± 11.9	1349.1 ± 15.7	-
	serum	281.8 ± 6.2	14.7 ± 0.7	9.8 ± 0.3	DET	-
Blank	inoculum **	DET	-	-	74 ± 2 *	-
		**Content (μg/g)**				
Rat 2	lung tissue	3.9 ± 0.3	-	-	-	-

**Table 2:** Distribution of bacterial (PvdE, Pch) and fungal (Fc, TafC) siderophores reflecting the respective *P. aeruginosa and A. fumigatus* coinfection. DET, detected (value between LOD and LOQ); -, not detected.

Sample		Concentration (ng/mL)			
	Specimen	PvdE	Pch	Fc	TafC
Patient 1	condensate	85.3 ± 3.4	135.1 ± 4.2	-	-
	urine	-	-	-	23 ± 1
Rat 3	urine	234 497 ± 13 961	69 460 ± 23 125	5 761 ± 191	1854 ± 49
Rat 4	urine	88 513 ± 2 553	20 711 ± 3474	-	-
Rat 5	urine	-	-	21 138 ± 2935	1 818 ± 124
Rat 6	urine	179 916 ± 16 104	66 965 ± 14 432	DET	564 ± 133
Rat 7	urine	108 313 ± 8 036	20 246 ± 2 609	DET	378 ± 14

## Supplementary Materials

The following are available online at https://www.mdpi.com/article/10.3390/jof7090730/s1, Figure S1: *P. aeruginosa* biomarkers in the lower airways. Figure S2: Pyoverdine quantitation plots in urine, serum and lung tissue homogenate. Figure S3: Quantitation of Pch, HHQ, and TafC in human/rat samples. Figure S4: Quantitation of Fc, TafC, Pvd, and Pch in the mixed infection model. Figure S5: Degradation of PvdE and HHQ during storage for a week at 37 °C. Figure S6: Fragmentation spectra of standard HHQ and PQS. Figure S7: Clinical observations made during the hospitalization of Patient 1. Figure S8, Monitoring *P. aeruginosa* in human urine along with fluconazole and its glucuronide.

## References

[1] Janbon G., Quintin J., Lanternier F., d’Enfert C. (2019). Studying fungal pathogens of humans and fungal infections: Fungal diversity and diversity of approaches. Genes Immun..

[2] Köhler J.R., Casadevall A., Perfect J. (2014). The spectrum of fungi that infects humans. Cold Spring Harb. Perspect. Med..

[3] Salmanton-García J., Sprute R., Stemler J., Bartoletti M., Dupont D., Valerio M., Garcia-Vidal C., Falces-Romero I., Machado M., de la Villa S. (2021). COVID-19–associated pulmonary aspergillosis, March–August 2020. Emerg. Infect. Dis..

[4] Verweij P.E., Rijnders B.J.A., Bruggemann R.J.M., Azoulay E., Bassetti M., Blot S., Calandra T., Clancy C.J., Cornely O.A., Chiller T. (2020). Review of influenza-associated pulmonary aspergillosis in ICU patients and proposal for a case definition: An expert opinion. Intensive Care Med..

[5] Shortridge D., Gales A.C., Streit J.M., Huband M.D., Tsakris A., Jones R.N. (2019). Geographic and Temporal Patterns of Antimicrobial Resistance in *Pseudomonas aeruginosa* Over 20 Years From the SENTRY Antimicrobial Surveillance Program, 1997–2016. Open Forum Infect. Dis..

[6] Curran C.S., Bolig T., Torabi-Parizi P. (2018). Mechanisms and targeted therapies for *Pseudomonas aeruginosa* lung infection. Am. J. Respir. Crit. Care Med..

[7] Cassini A., Högberg L.D., Plachouras D., Quattrocchi A., Hoxha A., Simonsen G.S., Colomb-Cotinat M., Kretzschmar M.E., Devleesschauwer B., Cecchini M. (2019). Attributable deaths and disability-adjusted life-years caused by infections with antibiotic-resistant bacteria in the EU and the European Economic Area in 2015: A population-level modelling analysis. Lancet Infect. Dis..

[8] Sass G., Nazik H., Penner J., Shah H., Ansari S.R., Clemons K.V., Groleau M.-C., Dietl A.-M., Visca P., Haas H. (2019). *Aspergillus-Pseudomonas* interaction, relevant to competition in airways. Med. Mycol..

[9] Novák J., Škríba A., Havlíček V. (2020). Cyclobranch 2: Molecular formula annotations applied to imzML data sets in bimodal fusion and LC-MS data files. Anal. Chem..

[10] Škríba A., Pluháček T., Palyzová A., Nový Z., Lemr K., Hajdúch M., Petřík M., Havlíček V. (2018). Early and non-invasive diagnosis of aspergillosis revealed by infection kinetics monitored in a rat model. Front. Microbiol..

[11] Petrik M., Umlaufova E., Raclavsky V., Palyzova A., Havlicek V., Pfister J., Mair C., Novy Z., Popper M., Hajduch M. (2021). 68Ga-labelled desferrioxamine-B for bacterial infection imaging. Eur. J. Nucl. Med. Mol. Imaging.

[12] Raymond K.N., Dertz E.A., Kim S.S. (2003). Enterobactin: An archetype for microbial iron transport. Proc. Natl. Acad. Sci. USA.

[13] Nazik H., Sass G., Ansari S.R., Ertekin R., Haas H., Deziel E., Stevens D.A. (2020). Novel intermicrobial molecular interaction: *Pseudomonas aeruginosa* Quinolone Signal (PQS) modulates *Aspergillus fumigatus* response to iron. Microbiology.

[14] Petřík M., Umlaufová E., Raclavský V., Palyzová A., Havlíček V., Haas H., Nový Z., Doležal D., Hajduch M., Decristoforo C. (2018). Imaging of *Pseudomonas aeruginosa* infection with Ga-68 labelled pyoverdine for positron emission tomography. Sci. Rep..

[15] Matthaiou E.I., Sass G., Stevens D.A., Hsu J.L. (2018). Iron: An essential nutrient for *Aspergillus fumigatus* and a fulcrum for pathogenesis. Curr. Opin. Infect. Dis..

[16] Sass G., Nazik H., Chatterjee P., Stevens D.A. (2021). Under nonlimiting iron conditions pyocyanin is a major antifungal molecule, and differences between prototypic *Pseudomonas aeruginosa* strains. Med. Mycol..

[17] Moura-Alves P., Puyskens A., Stinn A., Klemm M., Guhlich-Bornhof U., Dorhoi A., Furkert J., Kreuchwig A., Protze J., Lozza L. (2019). Host monitoring of quorum sensing during *Pseudomonas aeruginosa* infection. Science.

[18] Allegretta G., Maurer C.K., Eberhard J., Maura D., Hartmann R.W., Rahme L., Empting M. (2017). In-depth profiling of mvfr-regulated small molecules in *Pseudomonas aeruginosa* after quorum sensing inhibitor treatment. Front. Microbiol..

[19] Bassetti M., Azoulay E., Kullberg B.J., Ruhnke M., Shoham S., Vazquez J., Giacobbe D.R., Calandra T. (2021). EORTC/MSGERC definitions of invasive fungal diseases: Summary of activities of the intensive care unit working group. Clin. Infect. Dis..

[20] An J., McDowell A., Kim Y.-K., Kim T.-B. (2021). Extracellular vesicle-derived microbiome obtained from exhaled breath condensate in patients with asthma. Ann. Allergy Asthma Immunol..

[21] Škríba A., Patil R.H., Hubáček P., Dobiáš R., Palyzová A., Marešová H., Pluháček T., Havlíček V. (2020). Rhizoferrin glycosylation in *Rhizopus microsporus*. J. Fungi.

[22] Luptáková D., Pluháček T., Petřík M., Novák J., Palyzová A., Sokolová L., Škríba A., Šedivá B., Lemr K., Havlíček V. (2017). Non-invasive and invasive diagnoses of aspergillosis in a rat model by mass spectrometry. Sci. Rep..

[23] Singer M., Deutschman C.S., Seymour C.W., Shankar-Hari M., Annane D., Bauer M., Bellomo R., Bernard G.R., Chiche J.-D., Coopersmith C.M. (2016). The third international consensus definitions for sepsis and septic shock (Sepsis-3). JAMA.

