# Supplementary material for: Noninvasive Combined Diagnosis and Monitoring of Aspergillus and Pseudomonas Infections: Proof of Concept
Source: J Fungi (Basel). 2021 Sep 6;7(9):730. doi: 10.3390/jof7090730 (PMC8471143; doi:10.3390/jof7090730)
Supplement: Supplementary file 1 [file jof-07-00730-s001.zip › jof-1327146-supplementary.pdf]

Journal of
Fungi

MDPI

Supplementary Material

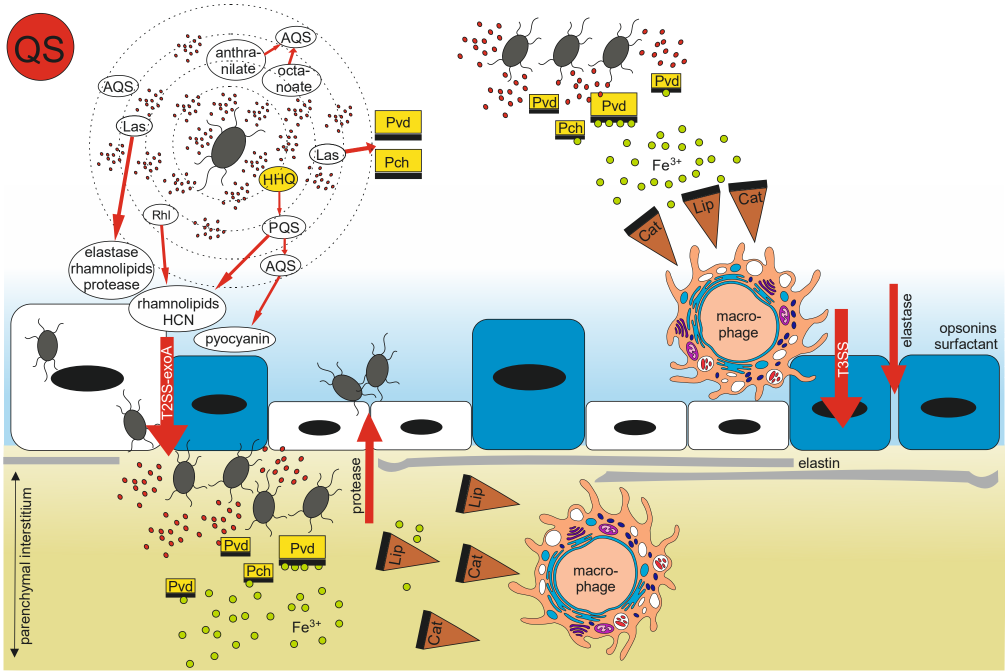

Figure S1. *P. aeruginosa* biomarkers in the lower airways. Type I (white) and Type II (blue) pneumocytes of the alveolar epithelium mediate gas exchange and secrete surfactant, respectively. If the alveolar epithelium, including elastin fibers, is damaged, *P. aeruginosa* can proliferate within type I or II pneumocytes. During infection, *P. aeruginosa* adheres to the epithelium and injects toxins through secretion systems (T2SS, T3SS). Activation of QS promotes accumulation of *P. aeruginosa* and production of QS molecules, including HHQ, the precursor of the *Pseudomonas* quinolone signal (PQS; 2-heptyl-3-hydroxy-4-quinolone). Efflux pump mechanisms release anthranilate, octanoate, and alkyl-quinolone signal (AQS) molecules. PQS is believed to provide an additional regulatory link between the Las and Rhl synthase enzymes involved in virulence and infection. Other autoinducer systems generate exotoxin A (exoA), hydrogen cyanide (HCN), reactive oxygen species, proteases, and elastases, all of which are needed for surfactant and opsonin decomposition in the lung epithelium. Microbial siderophores (Pvds, Pch) and other iron uptake systems compete with host iron transporters—small mammalian catechols (Cats) and proteinaceous lipocaline (Lip)—for extra- and intra-cellular iron. As 'stealth siderophores', Pvds may evade Lip recognition.

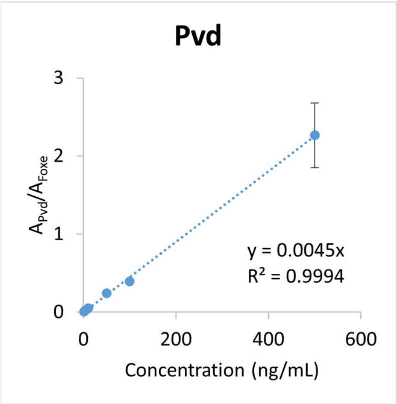

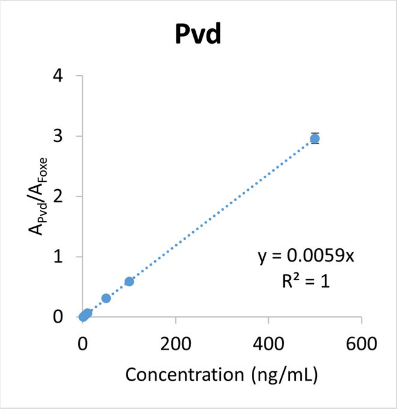

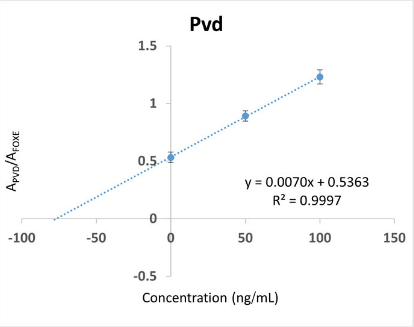

Figure S2. Pyoverdine quantitation plots in urine, serum, and lung tissue homogenate. Pvd quantitation in human/rat urine. A Pvd calibration curve was constructed from [M+Fe-H]2+ ion signals generated by electrospray ionization of a standard PvdE/PvdD mixture spiked into human urine. No sodium or potassium adducts were formed. Assuming the same ionization efficiency in the real samples, the whole Pvd signal was redistributed to other Pvd analogs produced by nonribosomal synthesis. Pvd was recorded in its protonated form. The instrumental LOD and LOQ were 1.1 ng/mL and 3.3 ng/mL, respectively. Pvd quantitation in human/rat serum. The instrumental LOD and LOQ were 0.4 ng/mL and 1.2 ng/mL, respectively. The data obtained can be found in the attached XLS spreadsheet. Pvd quantitation in rat 2 lung tissues with two quantified additions. The additions to rat 2 lung tissues were 50 and 100 ng/mL, respectively. The data obtained can be found in the attached XLS spreadsheet.

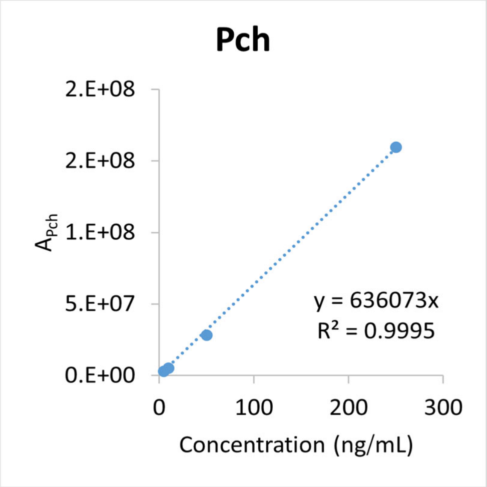

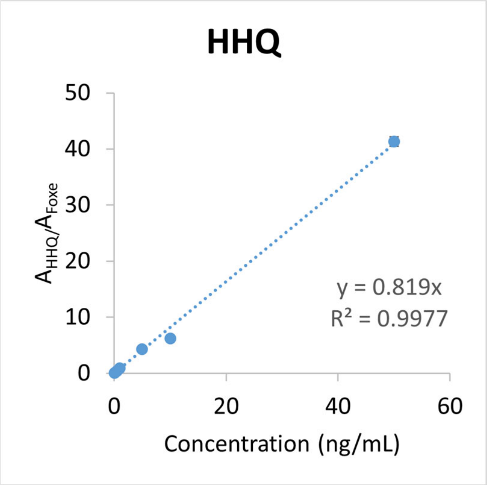

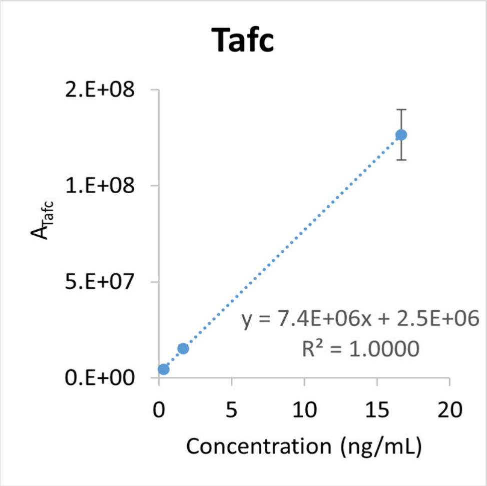

Figure S3. Quantitation of Pch, HHQ, and TafC in human/rat samples. Pch (blank, 5, 10, 50, 250 ng/mL) and HHQ (blank, 0.1, 0.5, 1, 5, 10, 50 ng/mL) calibration curves were constructed from $[M+H]^+$ ion signals. TafC calibration (blank, 0.3, 1.7, 16.7 ng/mL) was constructed from the sum of $[M+H]^+$, $[M+Na]^+$, and $[M+K]^+$ ion signals. All ions were generated by electrospray ionization of a standard solution spiked into the human urine. The instrumental LOD (2.10, 0.06, 0.27 ng/mL) and LOQ (6.37, 0.19 and 0.83 ng/mL) as well as original data can be found in the XLS spreadsheet to be provided on request.

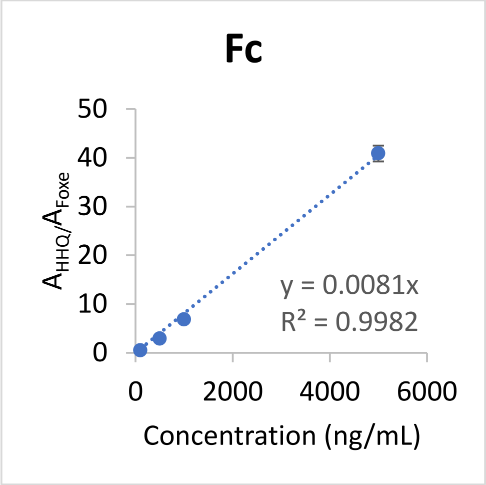

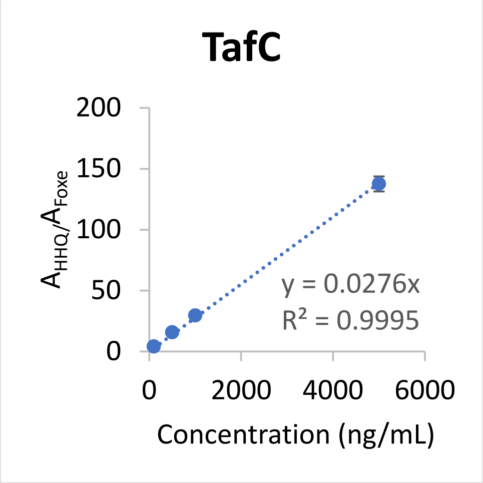

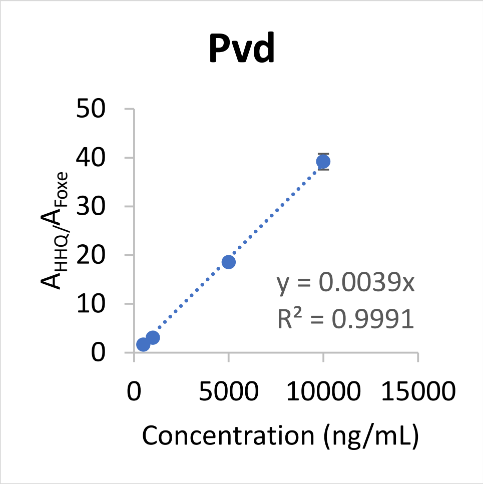

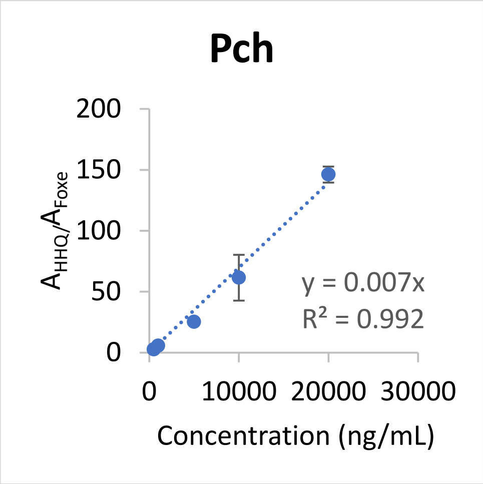

Figure S4. Quantitation of Fc, TafC, Pvd and Pch in the mixed infection rat model. FC (blank, 100, 500, 1,000, 5,000, 10,000 ng/mL), TafC (blank, 100, 500, 1,000, 5,000, 10,000 ng/mL), Pvd (blank, 500, 1,000, 5,000, 10,000, 20,000 ng/mL) and Pch (blank, 100, 500, 1,000, 5,000, 10,000, 20,000 ng/mL) calibration curves were constructed from $[M+H]^+$ ion signals. All ions were generated by electrospray ionization of a standard solution spiked into the rat urine. The instrumental LOD (161, 30, 313, 270 ng/mL) and LOQ (488, 93, 947, 817 ng/mL) as well as original data can be found in the XLS spreadsheet to be provided on request.

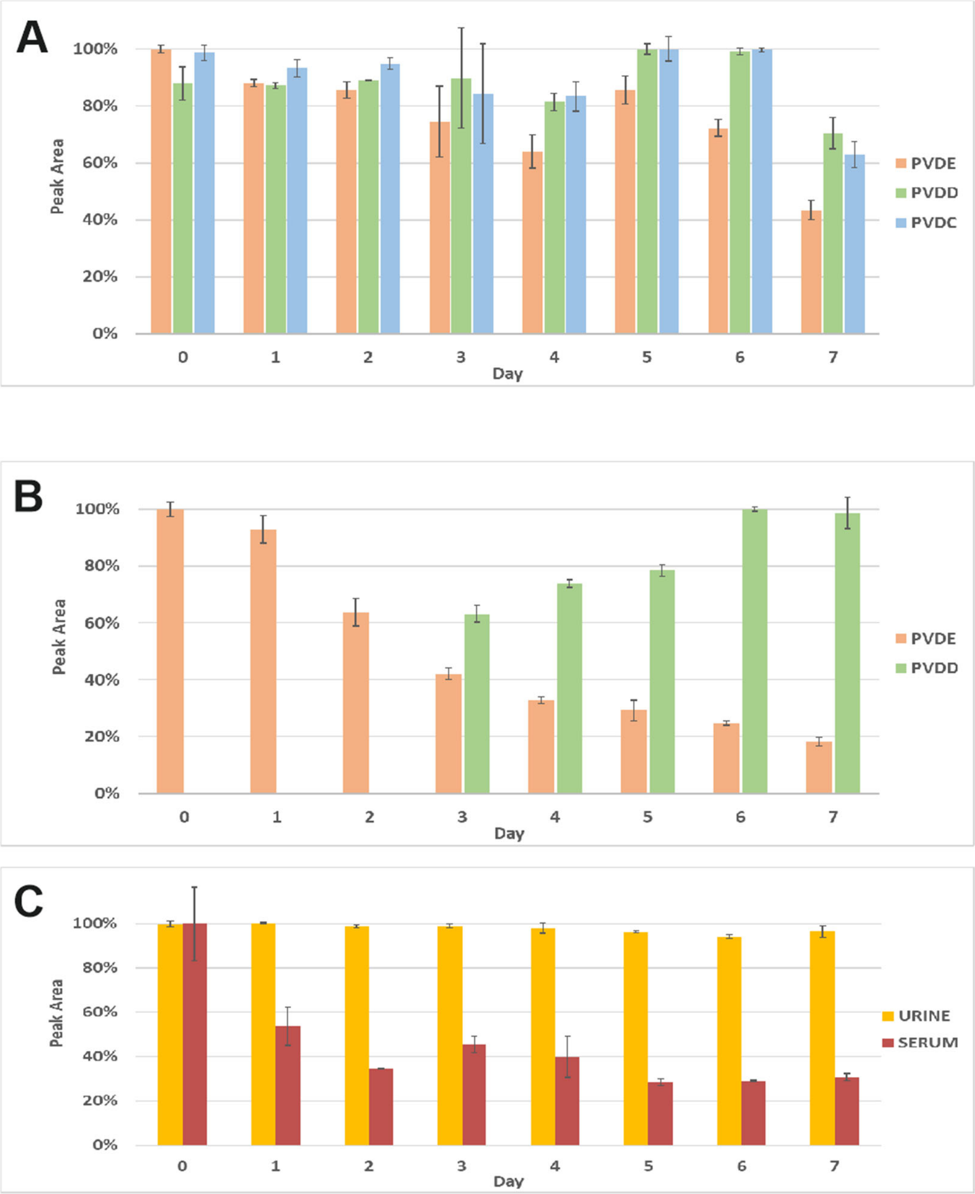

Figure S5. Degradation of PvdE and HHQ during storage for a week at 37 °C**.** PvdE was stored at 37 °C in a model serum (top panel, **A**) and urine from Patient 1 (middle panel, **B**). It should be noted that on the sample collection day no PvdD was detected in the patient's urine. PvdD appeared, as a PvdE degradation product, in the urine from the third day of storage onwards. The bottom panel (**C**) shows degradation of HHQ in a model serum and urine during a week of storage at 37 °C. Three technical replicates were collected by mass spectrometry.

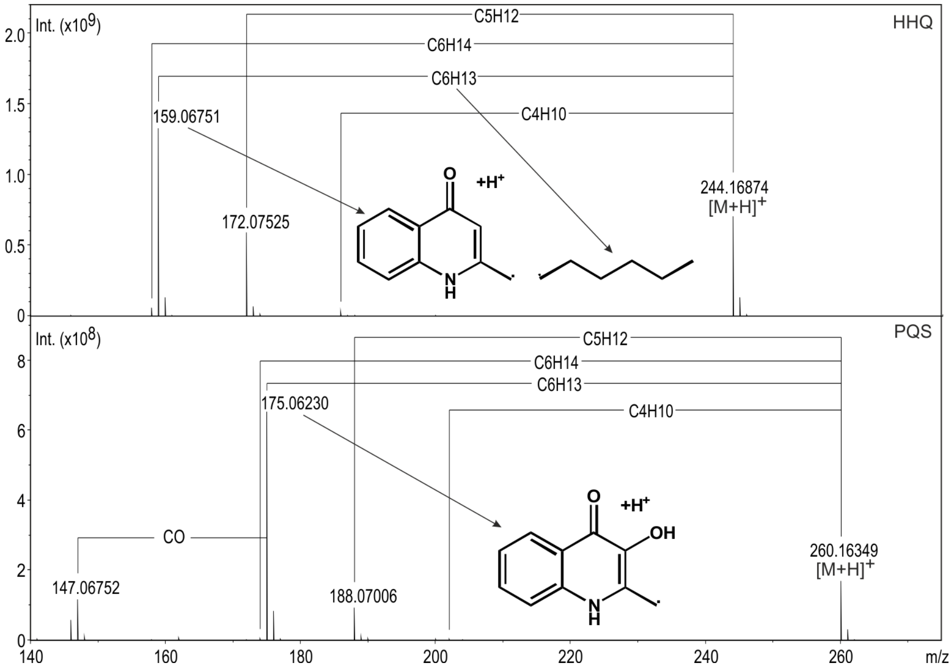

Figure S6. Fragmentation spectra of standard HHQ and PQS. Int. stands for the absolute ion intensity. Chemical formulas in the Figure, e.g., C5H12, indicate the neutral eliminated parts of the corresponding molecular structures during the fragmentation. The major fragment ion peaks arising from the corresponding protonated molecules was assigned to the quinolone core. HHQ and PQS stand for 2-heptyl-4-quinolone and 2-heptyl-3-hydroxy-4-quinolone, respectively.

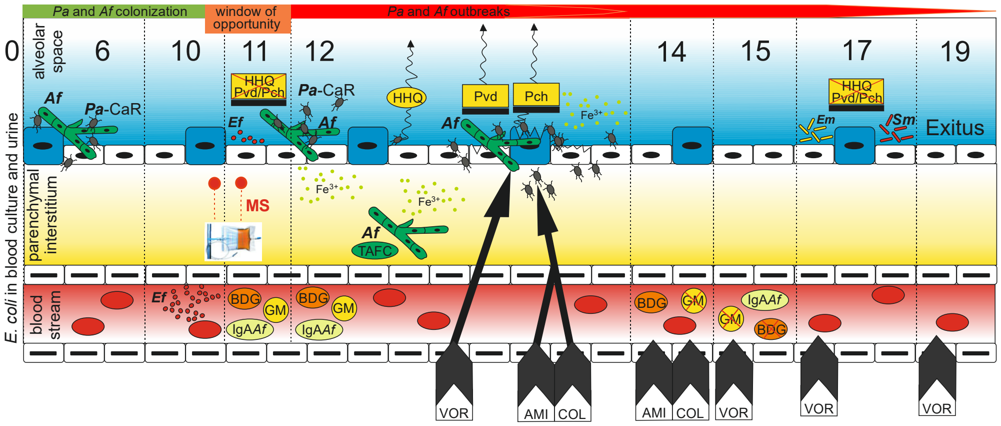

Figure S7. Clinical observations made during the hospitalization of Patient 1. Day 0: Blood culture was positive for *E. coli*. Day 6: carbapenem-resistant *P. aeruginosa* (*Pa*-CaR) with amikacin (AMI) and colistin (COL) susceptibility and *A. fumigatus* (*Af*) were cultured from sputum, appearing as colonies. Day 10: *E. faecium* (*Ef*) was found in blood culture. Days 10-11: Window of opportunity suggested for noninvasive mass spectrometry sampling. Day 12: *Af*, *Pa*-CaR, and *Ef* were detected in sputum by culture; HHQ, Pch, and Pvd were detected by mass spectrometry in breath condensate; TafC was detected in the urine; and elevated GM and BDG levels in the serum. On the same day, the serum had a positive IgA*Af* index, and an intensive antibiotic and antimycotic intervention began. Day 14: GM levels decreased in the serum during voriconazole (VOR) treatment. Day 15: The GM index became negative, but not the IgA*Af* index. Day 17: After seven days of amikacin, colistin, and VOR treatment, the cultured sputum was negative for both *P. aeruginosa* and *A. fumigatus*. Similarly, breath condensate and urine were negative for Pvd, Pch, and HHQ. Sputum was positive for *E. miricola* (*Em*) and *S. marcescens* (*Sm*) by culture.

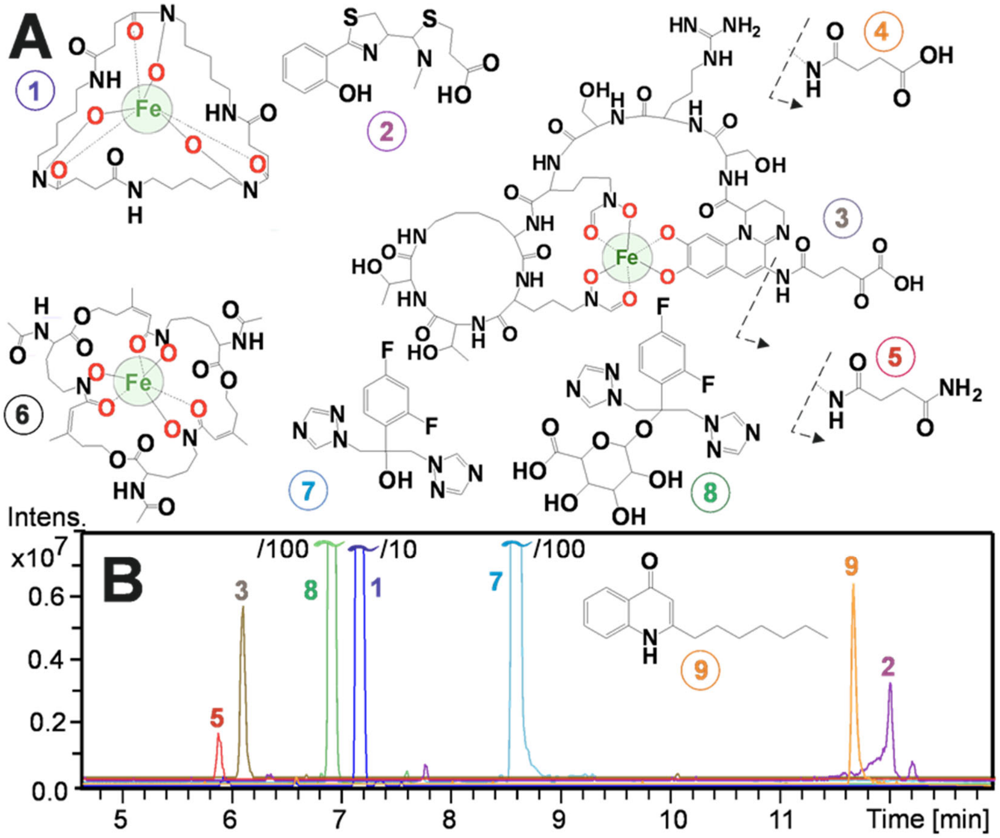

No.	Compound	Formula (M)	Ret. time	m/z calc.	Ionic form
1	FoxE	$C_{27}H_{48}N_6O_9$	7.2	654.2670	$[M+Fe-2H]^+$
2	Pch	$C_{14}H_{16}N_2O_3S_2$	12.0	325.0675	$[M+H]^+$
3	PvdC	$C_{56}H_{83}N_{17}O_{23}$	6.1	708.2555	$[M+Fe-H]^{2+}$
4	PvdD	$C_{55}H_{83}N_{17}O_{22}$	6.0	694.2579	$[M+Fe-H]^{2+}$
5	PvdE	$C_{55}H_{84}N_{18}O_{21}$	5.9	693.7659	$[M+Fe-H]^{2+}$
6	TafC	$C_{39}H_{60}N_6O_{15}$	8.8	906.3305	$[M+Fe-2H]^+$
7	FLC	$C_{13}F_2H_{13}N_6O$	8.6	307.1113	$[M+H]^+$
8	*N*-glucuronide-FLC	$C_{19}F_2H_{20}N_6O_7$	6.9	483.1434	$[M+H]^+$
9	HHQ	$C_{16}H_{21}NO$	11.7	244.1696	$[M+H]^+$

Figure S8. Monitoring *P. aeruginosa* in human urine along with fluconazole and its glucuronide. A: Chemical structures of siderophores PvdC (3), PvdD (4), PvdE (5), Pch (2), TafC (6), and FoxE (1) internal standard (IS), fluconazole (FLC, 7) and its glucuronide (8), and HHQ (9). Compound numbering is described in the associated table. B: Patient 2's urine profile compiled from reconstructed ion chromatograms recorded in low and high-mass experiments. Note the low chemical background noise when the 0.005 Da window was selected for ion chromatogram extraction. The absolute ion intensities (y axis, arbitrary units) provide rough estimates of electrospray ionization efficiencies for all analytes. In Patient 2's urine, no PvdD or TafC was detected.
